# Nursing Interventions to Prevent Complications in Patients with Peripherally Inserted Central Catheters: A Scoping Review

**DOI:** 10.3390/jcm14010089

**Published:** 2024-12-27

**Authors:** Andreia Vilão, Cidália Castro, Júlio Belo Fernandes

**Affiliations:** 1 Department of Nursing, Almada-Seixal Local Health Unit, 2805-267 Almada, Portugal; 2 Nurs * Lab, 2829-511 Almada, Portugal; jfernandes@egasmoniz.edu.pt; 3 Egas Moniz Center for Interdisciplinary Research (CiiEM), Egas Moniz School of Health & Science, 2829-511 Almada, Portugal

**Keywords:** PICC, nurse, prevention, complications

## Abstract

**Background**: A Peripherally Inserted Central Catheter (PICC) is a safe and effective Central Vascular Access Device when properly used. Therefore, it has become an increasingly frequent procedure. Nurses are often the professionals responsible for its insertion, maintenance, and removal. Despite the advantages of this device, it presents risks and possible associated complications. This scoping review aims to identify and analyze nursing interventions to prevent complications in adults with PICC. **Methods**: The review was conducted according to Joanna Briggs Institute’s scoping review proposal. The electronic databases Pubmed, CINAHL Complete, MEDLINE Complete, Cochrane Central Register of Controlled Trials, Nursing & Allied Health Collection: Comprehensive, Cochrane Database of Systematic Reviews, Cochrane Methodology Register, Library, Information Science & Technology Abstracts, and MedicLatina were consulted in October 2023. Additionally, we searched the websites of the Registered Nurses Association of Ontario and the Canadian Vascular Access Association. We included articles published in English and Portuguese between 2018 and 2023. **Results**: A total of 170 articles were initially identified. After selecting and analyzing the articles, 13 studies were included. This review identified nursing interventions in adults to prevent PICC-related complications, categorized into five main groups: pre-procedure, during the procedure, post-procedure, maintenance, and team management interventions. Nurses are pivotal in averting PICC complications by employing evidence-based nursing interventions at each process stage. **Conclusions**: The importance of nursing interventions in enhancing patient safety, improving health outcomes, and informing clinical practice highlights the need for standardized protocols, specialized training, and consistent patient education for PICC care.

## 1. Introduction

The insertion and maintenance of a vascular access device (VAD) for intravenous treatment is one of the most common procedures nurses perform in hospital settings [1]. However, most VADs are removed due to access-related complications [2,3].

A central vascular access device (CVAD) inserted into a peripheral or centrally located vein, with the tip located in the superior or inferior vena cava, is indicated when adequate peripheral access is not available, the osmolarity of the solution to be administered is greater than 900 mOsm/L (e.g., parenteral nutrition) or perfusion of a vesicant solution is required [4].

The use of a peripherally inserted central catheter (PICC), a CVAD inserted through a vein of an upper extremity and advanced to the distal third of the superior vena cava [5], has been increasing since it is considered safe and effective when properly used [6].

PICCs provide significant advantages. These include insertion under local anesthesia at the patient’s bedside, reducing patient discomfort by avoiding multiple venipunctures, contributing to the preservation of the peripheral venous system, ensuring safe administration of antibiotics and prolonged parenteral nutrition, providing excellent access for chemotherapy, longer indwelling time, lower risk of contamination, and widespread applicability across the continuum of care [5]. In addition, according to the same authors, it prevents iatrogenic complications such as pneumothorax and haemothorax, as the catheter is peripherally inserted.

However, PICCs have risks, including deep venous thrombosis, pulmonary embolism, bloodstream infection associated with the catheter, and PICC occlusion [7,8]. Occlusion is one of the most frequent complications of PICC, and it can cause treatment delays, increased costs, and premature catheter removal [9]. In addition, there is an association between PICC occlusion and central catheter-associated bloodstream infection [10]. When a clot forms and blocks the catheter, it can lead to bacterial proliferation, which can cause a significant increase in morbidity, mortality, and costs [11].

Presently, a range of healthcare professionals are implicated in the insertion and management of VADs, including PICCs, by the varying scopes of practice of professionals across the globe [4,10,12]. However, this may result in fragmented patient care [12]. Mussa et al. [13] identified notable differences in the composition of vascular access teams across several European countries, including Denmark, France, and Italy. These teams include a variety of professionals, such as nurses, anesthesiologists, and surgeons. Conversely, these teams are composed exclusively of nurses in the United Kingdom and Spain. Krein et al. [14] identified that most hospitals in the United States, specifically those with over 50 beds, maintain a specialized team of nurses proficient in PICC insertion and management.

Global variations in PICC-related nursing practices reflect differences in healthcare systems, resource availability, professional training, and regulatory standards. In North America, PICC insertion and care are frequently the responsibility of dedicated vascular access teams composed primarily of registered nurses with specialized training. These teams ensure adherence to standardized protocols, which has been associated with improved patient outcomes and reduced complication rates [15,16]. In contrast, European countries exhibit greater variability. While countries like the United Kingdom recognize nurses as professionals responsible for PICC insertion, other regions rely on multi-disciplinary teams where nurses, physicians, and anesthesiologists collaborate [17]. This diversity highlights the need for global harmonization of standards to ensure consistency in patient care and safety.

In low-resource settings, limited access to training, equipment, and evidence-based guidelines presents significant challenges to effective PICC management. Additionally, regional disparities in nurse education programs impact the preparedness of healthcare professionals [18].

In Portugal, it was only recently that interventions such as inserting, maintaining, and removing PICC lines were recognized as falling within the scope of nursing practice. The Board of Portuguese Nurses emphasizes the need for nurses receiving certified training to perform this procedure. It stresses the importance of healthcare institutions establishing protocols to ensure the necessary skills and knowledge for safe execution [19]. Consequently, nurses are willing to enhance their competencies in this field. In 2016, the Portuguese Association of Vascular Access (APoAVa) was founded to promote the development of skills in this specific domain through advanced training of physicians and nurses.

Emerging technologies and innovations have the potential to enhance nursing interventions related to PICCs significantly. For instance, using ultrasound guidance for PICC insertion has become a critical tool, improving the success rate and reducing complications [20]. Additionally, innovations such as near-infrared vein visualization devices [21] and antimicrobial-impregnated catheters are being developed to optimize patient safety and outcomes further [22]. Integrating digital platforms for real-time monitoring and electronic documentation can also streamline PICC care, enabling more precise and efficient intervention [23].

In a comprehensive review, Raynak et al. [24] sought to evaluate nurses’ current knowledge regarding the routine care and maintenance of VADs in adults. Their conclusion highlighted the need for enhanced educational preparation and workplace training for nurses in this area. Furthermore, the World Health Organization (WHO) has emphasized that the costs associated with adverse events far exceed those related to preventive measures to enhance the safety and quality of care [25].

Thus, this research holds considerable relevance as it supports nurses in making evidence-based decisions, facilitating the proper maintenance of PICC, and preventing related complications. This, in turn, ensures the effectiveness of treatment and upholds the quality of care provided.

This scoping aims to map and analyze the available scientific evidence on nursing interventions to prevent complications in patients with PICC.

## 2. Methodology

This scoping review follows the methodological approach proposed by the Joanna Briggs Institute (JBI) [26]. The study leveraged the Preferred Reporting Items for Systematic Reviews and Meta-Analyses Extension for Scoping Reviews (PRISMA-ScR) to augment comprehension, fortify transparency, and uphold the overall quality of the review.

The following research question was formulated using the acronym PCC (Population, Concept, and Context): “What interventions are used by nurses (P) to prevent complications (C) in adults with PICC (C)?”

### 2.1. Criteria for Inclusion

According to the methodology proposed by the JBI, inclusion and exclusion criteria were defined based on participants, concept, and context (PCC) (Table 1).

We selected all relevant published and unpublished articles with available abstracts and full texts as information sources. The study included articles published in English and Portuguese, as the reviewers were proficient in these languages. A time limit of 2018 to 2023 was set to prioritize recent articles that can be applied in practice based on the best available scientific evidence.

### 2.2. Search Strategy

A comprehensive search was conducted on PubMed (*n* = 110) and EBSCOHost search engine (*n* = 58), encompassing the databases CINAHL Complete, MEDLINE Complete, Cochrane Central Register of Controlled Trials, Nursing & Allied Health Collection: Comprehensive, Cochrane Database of Systematic Reviews, Cochrane Methodology Register, Library, Information Science & Technology Abstracts, and MedicLatina. In addition, we searched the websites of the Registered Nurses Association of Ontario (RNAO) (*n* = 1) and the Canadian Vascular Access Association (CVAA) (*n* = 1). The search yielded 170 articles in total. The final survey was completed on 19 October 2023.

In conjunction with Boolean operators, indexed medical subject headings were employed to formulate the following search strings:

- EBSCOhost search engine: [(PICC) OR (peripherally inserted central catheter) OR (PICC line) OR (catheterization, peripheral central venous)] AND [(adults) OR (adult) OR (aged) OR (elderly)] AND [(prevention) OR (intervention) OR (treatment) OR (program) OR (control) OR (strategy) OR (management) OR (protocol) OR (guideline) OR (policy) OR (catheter-related complications) OR (complications) OR (adverse effects)] AND (nurs*)
- PubMed database: [(PICC) OR (peripherally inserted central catheter) OR (PICC line) OR (catheterization, peripheral central venous)] AND [(adults) OR (adult) OR (aged) OR (elderly)] AND [(prevention) OR (intervention) OR (treatment) OR (program) OR (control) OR (strategy) OR (management) OR (protocol) OR (guideline) OR (policy) OR (catheter-related complications) OR (complications) OR (adverse effects)] AND [(nurse) OR (nurse interventions) OR (nursing care)].

### 2.3. Study Selection

Following the above inclusion criteria to the databases, the studies were screened by two independent researchers to ensure that only articles meeting the previously defined inclusion criteria were included. The selection process, which involved eliminating duplicate articles, was conducted in three stages. The initial stage of the review process entailed examining the article titles, followed by a review of the abstracts. Finally, the articles were read in their entirety. Any discrepancies were resolved through discussion or consultation with a third reviewer. The PRISMA-ScR presents this process, illustrated in a flowchart [27].

### 2.4. Data Extraction

To ensure a clear and comprehensive summary of the findings consistent with the scoping review’s aim and focus [26], two researchers independently conducted data extraction using a custom tabular tool developed to facilitate the organization and analysis of the data.

This tool includes the following information: authors, publication year, article title, country, study objective, study type, and nursing interventions/categories.

## 3. Results

**Figure 1:**
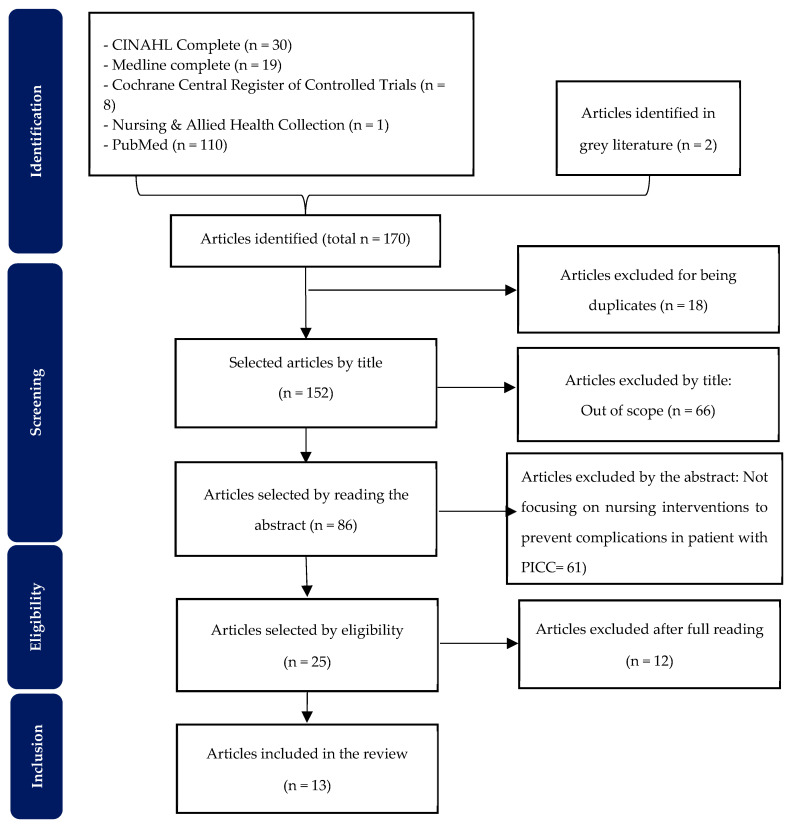
PRISMA flow chart for study selection.

This scoping review aimed to comprehensively outline the evidence in the literature on nursing interventions designed to prevent complications associated with PICC. Initially, a thorough search of databases yielded 168 articles, supplemented by two documents from grey literature. Following meticulous screening, 13 studies were deemed suitable for inclusion in this review. The PRISMA flowchart illustrating this process is depicted in Figure 1.

Out of the 13 articles selected for review, five were from the United States of America (USA) [11,14,28,29] and four were from China [30,31,32,33]. Additionally, two studies were conducted in Canada [4,10], one in Colombia [34], and one in Spain [35].

The research encompassed the following categories of studies: one case study [31], one retrospective longitudinal descriptive study [34], one correlational descriptive study [11], one descriptive phenomenological qualitative study [33], one cross-sectional study [32], and one retrospective cross-sectional study [30]. In addition, the following studies were included: one randomized controlled study [35], one quasi-experimental study [28], one quantitative survey study with a random sample [14], one retrospective cohort study [29], two literature reviews [10,36], and one systematic literature review [4].

To expedite the analysis of the findings, the interventions identified in response to the research question were classified by the thematic analysis method delineated by Braun et al. [36]. This inductive method allows for identifying patterns and themes within qualitative data sets, and the resulting classifications are presented in Table 2.

The inductive analysis [37] of the nursing interventions organized them into five categories according to their applicability: pre-procedure, during the procedure, post-procedure, maintenance, and team management. The following section presents a comprehensive description of each category of interventions.

### 3.1. Pre-Procedure Interventions

Following the standards set forth by the RNAO [4], a systematic assessment of an individual before inserting a VAD is a fundamental principle of good practice. Therefore, this assessment should be conducted on all individuals who require a VAD before its insertion.

In a literature review, Gupta et al. [36] posit that conducting a comprehensive and meticulous assessment of the individual’s medical and surgical history can mitigate the incidence of technical challenges encountered during the PICC insertion procedure and subsequent complications. Feng et al. [31] concurs with the position stated by Gupta et al. [36] that this is a prophylactic measure against the mispositioning of the catheter. Such an approach enables identifying any existing or potential predisposition to altered vascular anatomy, particularly the lateral thoracic vein, which can become dilated in certain clinical conditions, thereby increasing the probability of PICC becoming dislodged. Moreover, Gupta et al. [36] suggest that examining imaging records before the insertion of the PICC can facilitate the identification of these variations in vascular morphology.

A comprehensive review of the individual’s vascular access history before the procedure enables the identification of an alternative extremity for those requiring repeated PICC placements, thereby reducing the risk of injury and stenosis [36].

To mitigate the risk of thrombosis associated with PICC, Chen et al. [30] recommend that the initial assessment be conducted with a particular focus on individuals with identified risk factors, including a history of smoking, antineoplastic therapy (carboplatin or docetaxel), elevated D-dimer levels, and selecting the appropriate size of the PICC to be inserted. Nurses play a pivotal role in this process, as they must adapt the size of the PICC to align with the individual’s treatment requirements and vascular system. This is particularly important, as larger catheters have been associated with an elevated risk of endothelial damage and vascular reactivity [4,30]. Gupta et al. [36] concur and posit that the size of the catheter should be less than 45% of the diameter of the vein where it will be inserted, with the utilization of PICCs with a diminished number of lumens being associated with a reduced risk of thrombosis.

To select the appropriate catheter size, it is essential to conduct a preliminary assessment of the individual’s vasculature using US [4,36]. This enables the mapping of available veins and the evaluation of their permeability, compressibility, and diameter. The basilic vein constitutes the optimal choice for the initial access attempt, as it can be readily identified in the medial region of the arm. The brachial vein may be considered the second most suitable option, while the cephalic vein is the least preferable due to its high anatomical variability and association with a higher incidence of thrombosis [36].

Patients with elevated D-dimer levels (>0.55 mg/L) require careful assessment, referral, and, if indicated, the initiation of prophylactic anticoagulant therapy [30]. Conversely, a review of the clinical history of coagulopathy or anticoagulant/antiplatelet therapy enables the identification of individuals at the highest risk of bleeding complications. This allows for the implementation of a preventive approach by optimizing the coagulation profile before the procedure [36]. Additionally, Gupta et al. [36] indicate that selecting a smaller gauge needle may reduce bleeding risk.

The selection of the PICC material is crucial to reducing the potential for infection. Gupta et al. [36] posit that utilizing antimicrobial PICCs can mitigate the risk of infection. This conclusion is supported by the study conducted by Kagan et al. [29], which demonstrated that the utilization of PICCs lacking antimicrobial impregnation represents a significant risk factor for catheter-associated bloodstream infection. The authors thus propose that using PICC catheters impregnated with antimicrobials should be considered for all individuals, not merely in high-risk settings.

The selection of the optimal site for PICC insertion is also a component of the pre-procedural assessment. Ying et al. [32] sought to investigate the influence of this decision on the incidence of catheter-related complications and the level of comfort experienced by the individual. Their findings indicated that inserting the PICC in non-dominant arms was associated with a reduced risk of complications, including catheter occlusion and bleeding at the insertion site, while providing a superior comfort level for the individual. Gupta et al. [36] assert that minimizing patient discomfort after the procedure is contingent upon avoiding placement of the access in the antecubital fossa.

Gupta et al. [36] propose that the PICC insertion procedure should be monitored using a checklist initiated in the pre-procedure phase and concluded in the post-procedure phase. This is proposed as a preventative measure for the occurrence of iatrogenic adverse events, such as retained guidewires.

### 3.2. During Procedure Interventions

Pain management during the PICC insertion procedure should be planned to ensure patient comfort and satisfaction and reduce fear and anxiety, particularly in cases where the patient exhibits a needle phobia [4]. In this regard, the RNAO [4] advises implementing non-pharmacological and pharmacological pain management strategies during catheter insertion, considering the individual’s preferences and past experiences. Gupta et al. [36] state that sedation is only indicated in adults with significant anxiety or cognitive impairment.

Regarding the actual insertion of PICC, as a means of guiding the puncture and reducing the risk of misplacement, Feng et al. [31] and Gupta et al. [36] recommend that the procedure be guided by real-time imaging using US and intracavitary EKG or fluoroscopy. According to these authors, this approach improves anatomical orientation and visualization of the surrounding structures. However, Feng et al. [31] suggest that when there is a history of dilation of the lateral thoracic vein, fluoroscopy is the preferred method, as this allows for continuous monitoring of the catheter throughout the procedure, enabling prompt identification of any displacement of the catheter tip.

Another intervention that allows the PICC to be correctly positioned at the cavo-atrial junction, which refers to the point where the superior vena cava meets and merges with the upper wall of the right atrium [10], consists of adapting the length of the catheter to the individual in whom it will be placed through specific measurements to be taken at the beginning of the procedure [36].

Once inserted, the PICC should be secured to the skin to prevent displacement. Adhesive stabilization devices like StatLock™ (Bard, Covington, GA, USA) or sutures may be employed [36]. Padilla-Nula et al. [35] propose the addition of cyanoacrylate glue to the fixation of the PICC in their randomized controlled study, citing a lower incidence of peri-catheter bleeding and exudation and a lower incidence of displacement.

### 3.3. Maintenance Interventions

Broadhurst et al. [10] stated that the frequency of catheter access should be limited to reduce the likelihood of occlusion. Brodnik et al. [11] also propose that the risk of PICC occlusion can be mitigated by flushing the lumens after each catheter use and more frequently if intravenous fluids are being administered continuously.

Moreover, Broadhurst et al. [10] propose that lumen flushing should be conducted with 0.9% sodium chloride through the pulsatile or “push-pause” technique, not only following each catheter utilization but also between the administration of incompatible drugs, thus preventing the formation of precipitation or residues that could potentially occlude the lumen. Suppose the administered therapy is incompatible with sodium chloride. In that case, a compatible solution should be used instead, such as dextrose in water, and then the lumen should be flushed once more with 0.9% sodium chloride [10].

As Gupta et al. [36] indicate, it is suggested that PICC lumens be heparinized to maintain their permeability. In certain clinical scenarios, Broadhurst et al. [10] propose that alternative lumen-blocking solutions, such as heparin, sterile ethanol, taurolidine, sodium citrate, and ethylenediamine tetraacetic acid (EDTA), may be considered following flushing with 0.9% sodium chloride. Nevertheless, potential risks must be considered, such as heparin-induced thrombocytopenia, which can occur at any dosage [10].

Observing aseptic precautions when handling PICC is a fundamental aspect of preventing PICC-related infection, as outlined by Gupta et al. [36]. Furthermore, utilizing an antimicrobial disk, such as the BioPatch^®^ protective disk impregnated with chlorohexidine, at the PICC insertion site has been demonstrated to diminish the likelihood of this adverse event [36].

When clinically indicated, the PICC can be safely removed without difficulty. However, the length of the removed PICC should be measured and compared with the measurement recorded at the time of insertion to rule out the possibility of rupture [36].

A systematic literature review conducted by the RNAO [4] indicates that providing health education to individuals and their families/caregivers regarding their VAD can reduce complications and enhance satisfaction and comfort regarding healthcare when involved in the process.

### 3.4. Post-Procedure Interventions

Following the completion of the PICC insertion procedure, it is imperative to confirm the location of the catheter tip in the cavo-atrial junction. If the procedure was performed with the assistance of fluoroscopy, the positioning would be confirmed via this modality [31,36]. Alternatively, as proposed by the same authors, intracavitary EKG can be employed as a highly accurate non-invasive method.

In the absence of imaging-based guidance during the PICC insertion procedure, radiographic post-procedure control is necessary to avoid potential complications, such as thrombus formation and catheter occlusion, vascular perforation, bleeding, and cardiac tamponade, as this allows for direct observation of the catheter’s path, the catheter’s tip location, and the catheter’s position, including whether it is misplaced, kinked, or if the catheter’s length is inadequate [10,31,36].

### 3.5. Team Management Interventions

Five authors [4,10,28,33,36] highlight the need for training healthcare professionals responsible for inserting, maintaining, and managing PICC lines to prevent potential complications associated with these catheters. Gupta et al. [36] propose that nurses who have undergone specialized training should conduct PICC insertion and maintenance. The entire nursing team should receive education on properly handling these catheters.

Bozaan et al. [28] implemented a multimodal strategy that included training all professionals involved in the PICC selection and insertion process on indications for appropriate PICC use and selection of the most appropriate PICC type. Implementing the single-lumen PICC as the standard device in the electronic prescription system and precisely defining the criteria for the proper use of the multi-lumen PICC resulted in a notable improvement in its appropriateness and decreased complications, including infection and catheter occlusion.

Broadhurst et al. [10] state that continuing education for healthcare professionals and validating their skills are guaranteed to prevent PICC occlusion. This must include training in the following areas: device permeabilization, assessment, prevention, and management of catheter occlusion; type of CVAD and additional characteristics; maintenance of records; ongoing surveillance and monitoring of results [10].

Zheng et al. [33] revealed that nurses who have yet to undergo specialization in the field of PICC demonstrate a more limited understanding of the prevention and management of drug-induced catheter occlusion. Moreover, the researchers determined that the paucity of time and communication breakdowns between nurses, the PICC specialist team, and the pharmaceutical professional contribute to an elevated incidence of this complication. The researchers thus conclude that targeted interventions to reduce the incidence of drug-induced PICC occlusion should encompass adjustments to the nurse-to-patient ratio, enhancements to the competencies of the nursing staff involved in catheter management, and improvements in communication between the various professionals involved in the process.

In 2021, the RNAO [4] recommended implementing practical education sessions on inserting and managing VADs. By their guideline, such sessions should encompass skills practice, supervised insertion and management of PICC, and practical training, which may include training in a high-fidelity simulation laboratory.

Implementing specialized nursing teams dedicated to vascular access appears to be an effective intervention strategy for preventing complications, as evidenced by three articles in the literature [4,14,34]. Cortés et al. [34] conducted a study to evaluate the outcome indicators of a nurse-led Specialized Vascular Access Program. The indicators assessed included the time to catheter placement, the appropriate use of PICCs, the percentage of US-guided catheter insertion, the percentage of successful first insertion attempts, the length of time the catheter remained in place, and the rate of associated infections. The program demonstrated positive outcomes in all of these indicators, thereby underscoring the significance of establishing teams of nurses with expertise in vascular access to enhance outcomes and improve the quality and safety of care.

Krein et al. [14] found that the provision of care by teams of nurses with specialized training in PICC placement is associated with a higher level of adherence to evidence-based practices for the prevention of catheter-related infection.

In its systematic review of the literature, the RNAO [4] recommends implementing vascular access specialist teams (VASTs) to support the insertion and management of VADs. This recommendation is based on the evidence that these teams can reduce the incidence of associated complications.

## 4. Discussion

This review presents a comprehensive overview of nursing interventions to prevent complications in patients with PICC.

The variety of articles from disparate countries may indicate that nurses worldwide are concerned about the pursuit of enhanced care to prevent complications associated with PICC, and efforts are ongoing in numerous countries worldwide.

Nurses implement interventions to prevent PICC-related complications at each stage of the process, including the planning, insertion, maintenance, and removal of the catheter.

Several authors have proposed that a comprehensive and systematic evaluation of the individual with an indication for PICC insertion is a crucial intervention, as it enables the identification of risk factors and previous medical or surgical conditions that could potentially result in complications [4,30,31,36].

A comprehensive initial assessment should encompass an evaluation of the available veins, recent and past history with vascular access, comorbidities that may influence the risk of infection or other complications such as hemorrhagic or thrombotic, the critical or chronic nature of the disease, the type and duration of treatment, future needs, specific access requirements, and the individual’s preferences, as these factors are integral to the determination of the level of risk and the optimal type, size, and insertion site of the device that best aligns with the individual’s needs [4,38,39,40]. This specific approach to assessing and selecting the VAD has resulted in successful placement with a low incidence of complications during and after the procedure, allowing for the preservation of the vascular anatomy and the continuation of treatment without interruption and with enhanced reliability [40,41].

Over the past two decades, many factors have contributed to improving the safety of the PICC insertion procedure. The most notable of these is the increasing utilization of US technology at various stages of the procedure [38].

As stated by Feng et al. [31] and Gupta et al. [36], using the US to guide the PICC insertion procedure represents a fundamental intervention. Incorrect catheter positioning can result in malfunction and an elevated risk of complications [42,43]. This is consistent with the recommendations of the American Institute of Ultrasound in Medicine (AIUM) [44] in its guidelines defining the criteria for using the US in guiding vascular access procedures. The AIUM guideline states that PICC insertion should be performed under US guidance, as this improves the vascular assessment technique, allows for the selection of an optimal vein for puncture, and increases the success rate of insertion while reducing complications, particularly the risk of thrombosis.

Brescia et al. [38] developed a safe PICC insertion protocol (SIP protocol), which integrates eight strategies to enhance the procedure’s safety, efficacy, and cost-effectiveness. Of the eight strategies, six employ the use of US, including the assessment of the vascular system before the procedure, the selection of the appropriate vein and catheter gauge, the identification of the median nerve and brachial artery, the guidance of the venipuncture, the monitoring of the catheter tip’s progression, and the assessment of the location of the PICC tip. Nevertheless, this approach does not eliminate the necessity for verifying the catheter tip’s position at the cavo-atrial junction through EKG guidance or, in the absence of this, by a chest X-ray by the recommendations set forth by Feng et al. [31], Gupta et al. [36], and Broadhurst et al. [10].

Another intervention identified as preventing further complications in this review is selecting the PICC gauge. A PICC catheter that occupies more than a third of the diameter of a blood vessel has been demonstrated to reduce blood flow and increase the risk of thrombosis [40]. As stated by Chen et al. [30] and the RNAO [4], the smallest gauge PICC that meets the individual’s needs and is adapted to their vasculature should be selected to reduce the risk of catheter-related thrombosis. Gupta et al. [36] posit that the catheter size should be less than 45% of the vein diameter. Conversely, Brescia et al. [38] argue that the ideal scenario is for the vein diameter to be at least three times greater than the catheter size, occupying only 33% of the diameter. This assessment should be performed using the US.

The utilization of sutures to secure the PICC has been associated with an increased risk of infection at the catheter insertion site, tissue damage, and catheter displacement [38]. Consequently, this approach has been discouraged. As an alternative, adhesive-based fixation devices, subcutaneous tissue anchoring, and cyanoacrylate glue with a transparent dressing to protect the skin have been demonstrated to reduce the risk of bleeding and bacterial contamination [35,38]. In the study conducted by Padilla-Nula et al. [35], a lower incidence of peri-catheter bleeding and exudation and a lower incidence of catheter displacement were observed when a sutureless device plus transparent dressing was used in comparison to the same fixation method plus the application of cyanoacrylate glue. However, no statistically significant differences were observed in the incidence of phlebitis or pain related to the catheter [35].

One critical area that requires particular attention in preventing complications is the management of biofilm formation on the catheter surface. Biofilm is recognized as a significant contributor to catheter-related bloodstream infections and other complications such as thrombosis and occlusion. Studies highlight the importance of implementing targeted strategies to prevent biofilm formation [45,46,47].

It is recommended that interventions to maintain the permeability of the PICC should be carried out regularly to prevent potential complications associated with the catheter’s use, namely occlusion, thrombosis, infection, and delays or interruptions in therapy administration [10].

The evidence suggests that the selection of single-lumen PICCs is associated with a lower risk of complications [28,40,48] and the implementation of aseptic precautions during catheter handling, which reduces the risk of infection [36]. Moreover, maintaining the permeability of the lumens through frequent flushing and whenever therapy is administered is a recommended intervention for preventing occlusion [10,11,49]. Nevertheless, the optimal frequency for these flushes has yet to be established, and the relevance of such a determination remains unclear [9,11].

In addition to the practical component of the procedure, the evidence indicates that continuous education and validation of the skills of healthcare professionals responsible for the care and management of PICCs represent key factors in preventing complications [4,10,28,33,36]. Although the US offers distinct advantages in assessing and inserting PICCs, it requires training and must be carried out by qualified professionals [40]. Similarly, the AIUM [44] states that nurses who perform this procedure should receive adequate training in using the US. This is consistent with the recommendations set forth by the RNAO [4] regarding the practical education of healthcare professionals, as evidence suggests that such education increases the number of successful insertion attempts, potentially reduces catheter-related complications, and may improve the professional’s confidence in performing the procedure. Furthermore, the importance of education extends beyond healthcare professionals to include patients themselves. Providing patients with education about their vascular access devices is a critical aspect of nursing care, as it not only empowers them with the knowledge to participate in their own care but also contributes to reducing complications. When patients are informed about proper care, maintenance, and potential signs of complications, such as infection or thrombosis, they are better equipped to adhere to care recommendations, recognize early warning signs, and seek timely intervention.

Therefore, teams of nurses who have undergone specialized training in vascular access to insert and manage these devices are proposed to produce indicators, reducing complications and, consequently, health costs [4,14,34]. Krein et al. [14] found that teams of nurses with expertise in PICC placement demonstrate a higher level of adherence to evidence-based practices, including using maximum barrier precautions, chlorhexidine disinfection of the insertion site, and using checklists during the insertion procedure. In their study, Kagan et al. [29] observed a lower incidence of PICC catheter-associated infection when placed by the nurse venous access team compared to interventional radiologists.

The professionals who compose these teams obtain knowledge and skills through formal training supported by scientific evidence, which enhances VAD success, reduces the number of insertion attempts, and mitigates associated complications such as infection and thrombosis [4,14,34,50,51,52] as a consequence of team dynamic and the meticulous manner in which they conduct their activities, consistently supported by the most advanced scientific evidence, with comprehensive educational and training programs that are integrated into their practice regularly and subject to periodic review [13,40,53]. Moreover, they provide training for other healthcare professionals within the institution, ensuring local practices align with the most current evidence-based standards [13,54]. Mussa et al. [13] propose that the optimization of care for individuals with VADs should be regarded as a distinct specialty, encompassing device selection, insertion, maintenance, and removal, based on the principle of selecting the right device for the right patient and the right treatment by the right professional.

Overall, this review highlights nurses’ significant role in preventing complications associated with PICC. However, nursing practices regarding PICC insertion and management vary across countries, influenced by factors such as healthcare system structures, professional regulations, and available resources [55,56]. In some countries, vascular access teams are composed exclusively of nurses, strongly emphasizing nurse specialization in this area. Conversely, in other nations, these teams often include a mix of healthcare professionals, suggesting differing scopes of practice and interprofessional dynamics.

The prevalence of specialized vascular access teams in hospitals demonstrates a systemic prioritization of PICC expertise. These teams are frequently led by nurses who have undergone advanced PICC placement and management training, underscoring the importance of standardized education and skill validation. Meanwhile, in countries like Portugal, PICC insertion within the nursing practice is relatively recent, requiring targeted training initiatives and institutional protocols to ensure safe and effective care.

The differences in training programs across countries can profoundly influence outcomes and patient safety. In regions where standardized, evidence-based training is widely available, nurses are better equipped with the necessary skills and knowledge to prevent PICC-related complications, such as infection or occlusion. In contrast, regions with limited access to such training may face higher complication rates, highlighting the critical link between education and patient safety.

Additionally, the availability of professional development opportunities varies widely. While some nations offer robust continuing education programs and active professional associations dedicated to vascular access, others may lack the infrastructure to support ongoing skill enhancement. This discrepancy affects the consistency of nursing care and the confidence and competence of nurses performing PICC-related procedures.

These variations may also stem from disparities in access to resources, such as ultrasound technology for guided PICC insertion, and differences in educational frameworks.

Understanding these global differences is essential for identifying best practices that can be adapted across diverse healthcare settings. Future efforts should focus on harmonizing PICC-related nursing practices through international collaborations, establishing universal guidelines, and sharing evidence-based strategies. Expanding access to high-quality training programs and fostering global knowledge exchange would enhance patient outcomes and promote a more cohesive and equitable approach to vascular access care worldwide.

### Limitations

This review provides significant value by identifying and detailing nurse-led interventions to prevent complications associated with procedures, such as nurse-performed PICC placement, serving as a guide for healthcare professionals. However, the review has certain limitations. Firstly, due to the nature of a scoping review, the interventions identified were not analyzed in terms of effectiveness. Secondly, the methodological choices regarding selecting databases, descriptors, temporal limits, and language restrictions to English and Portuguese may have introduced a language bias, potentially excluding relevant studies published in other languages. Additionally, the regional focus of the studies conducted in specific countries may have limited the generalizability of the findings. Variations in healthcare systems, professional roles, and nursing practices across different regions could influence the applicability of these interventions. Furthermore, the primary limitations identified in the studies, such as lack of standardized protocols, and methodological differences, may have also influenced the results. These factors should be considered when interpreting the conclusions of the review. Future research should include a broader linguistic and geographic scope to provide a more comprehensive understanding of PICC-related nursing practices.

## 5. Conclusions

This scoping review provides an overview of the current literature on nursing interventions to prevent complications associated with PICC. The review highlights the crucial role of nursing involvement at each stage of the process, from the initial planning stage to the actual removal of the PICC, in improving health outcomes and enhancing the quality and safety of care provided to individuals with PICC.

The stage preceding the PICC insertion procedure is critical in reducing the risk of complications associated with the catheter. This is contingent upon conducting a comprehensive and personalized assessment of the individual characteristics of the patient who requires a PICC. Furthermore, the incorporation of US at multiple stages of the procedure, intracavitary EKG for the positioning of the PICC tip, fixation without sutures, and cyanoacrylate glue enhances the safety profile and cost-effectiveness of the procedure. Several studies have indicated that a team-based approach to assessing, inserting, and maintaining VADs can improve clinical outcomes, patient experience, and healthcare processes, thereby enhancing the quality of care provided in vascular access within an organization.

Further research is suggested to enhance comprehension of the function, structure, and methodological aspects of specially trained teams designated for inserting and managing PICCs.

## Figures and Tables

**Table 1:** Inclusion/exclusion criteria.

Parameter	Inclusion Criteria	Exclusion Criteria
Population	Studies focusing on nurse-led interventions for individuals aged 18 and older.	Participants < 18 years old.
Concept	Studies that explore nursing interventions to prevent complications related to PICC.	Studies not focusing on the prevention of complications related to PICC. Studies that do not describe interventions conducted by nurses.
Context	Studies involving individuals with a PICC in place.	Studies involving individuals with other types of VAD.

**Table 2:** Data extraction and synthesis.

Author/Year/Title/Country	Aim	Study Design	Interventions/Categories
Feng et al. [31], 2020 A peripherally inserted central catheter misplacement into a lateral thoracic vein: A case report. China	Reporting a case of PICC misplacement into the lateral thoracic vein.	Case study	**Pre-procedure:** -Assess the person’s medical history to detect any existing or potential predisposition to dilation of the lateral thoracic vein. **During the procedure:** -Perform real-time image-guided catheterization using ultrasound (US) and intracavitary electrocardiogram (EKG) or fluoroscopy. **Post-procedure:** -Check the position of the PICC tip using fluoroscopy, intracavitary EKG, or chest X-ray.
Padilla-Nula et al. [35], 2023 Effectiveness of cyanoacrylate glue in the fixation of midline catheters and peripherally inserted central catheters in hospitalized adult patients: Randomized clinical trial. Spain	To evaluate the efficacy of using cyanoacrylate glue (CAG) as a means of fixing midlines and PICCs with the modified micro-Seldinger technique in hospitalized adult patients.	Randomized controlled trial	**During the procedure:** -Fix midlines and PICC with CAG to reduce the incidence of bleeding and peri-catheter exudation and displacement.
Cortés et al. [34], 2022 Evaluation of Indicators of a Vascular Access Device Program led by Nursing Professionals in a High-complexity University Hospital in Colombia. Colombia	Evaluate the result indicators of the Specialized Vascular Access Program led by nurses to structure improvement plans.	Descriptive longitudinal retrospective study	**Team management:** -Create Specialist Nursing Teams for PICC placement.
Ying et al. [32], 2020 Impact of arm choice for peripherally inserted central catheter (PICC) insertion on patients: a cross-sectional study. China	To explore the impact of the choice of arm (dominant or non-dominant) on the rate of catheter-related complications and the degree of comfort I patients with PICC.	Cross-sectional study	**Pre-procedure:** -Select the non-dominant arm for PICC insertion.
Chen et al. [30], 2020 Incidence and risk factors of symptomatic thrombosis related to peripherally inserted central catheter in patients with lung cancer. China	To determine the incidence and risk factors associated with PICC-related thrombosis (PICC-RT) in lung cancer patients.	Retrospective cross-sectional study	**Pre-procedure:** -Conduct a complete initial assessment of the patient to detect risk factors for developing PICC-RT, such as smoking, treatment with antineoplastic drugs (carboplatin or docetaxel) and high D-Dimers. -Select the smallest size PICC according to the patient’s predisposing factors. -Assess the need for prophylactic anticoagulant therapy in patients with high D-dimer levels.
Bozaan et al. [28], 2019 Less Lumens-Less Risk: A pilot intervention to increase the use of Single-Lumen peripherally inserted central catheters. USA	Increase the use of single-lumen PICCs in hospitalized patients to reduce the risk of complications.	Quasi-experimental study	**Team management: ** -Provide training to all professionals involved in the process (including indications for the appropriate PICC use and how to select the most clinically appropriate features, such as the number of lumens and the catheter gauge). -Implement the single-lumen PICC as the standard device in the electronic prescription system for PICC placement. -Define criteria for the appropriate use of multi-lumen PICCs.
Zheng et al. [33], 2020. Nurses’ knowledge of the management of drug-induced peripherally inserted central catheter obstruction: A descriptive phenomenological study. China	To describe and understand nurses’ level of knowledge about the management of drug-induced PICC occlusion. To identify why nurses are unaware of/do not use the management techniques for drug-induced PICC occlusion recommended by the Infusion Therapy Standards of Practice.	A qualitative descriptive phenomenological study	**Team management:** -Adjust the nurse-to-patient with PICC ratio. -Train nurses on drug-induced PICC occlusion. -Improve the communication process between the nurses on the ward and the nurses on the PICC management team. -Encourage communication between nurses and pharmacists in managing the therapy to be administered to the patient with a PICC.
Kagan et al. [29], 2018 Peripherally inserted central catheter-associated bloodstream infection: Risk factors and the role of antibiotic-impregnated catheters for prevention. USA	To evaluate the risk factors for PICC-associated bloodstream infection with a focus on the effect of antimicrobial-impregnated versus non-impregnated PICCs.	Retrospective cohort study	**Pre-procedure:** -Preferably select PICCs impregnated with antimicrobials.
Brodnik et al. [11], 2023 PICC Line Occlusions: Implications and Opportunities for Medical-Surgical Nurses. USA	To identify factors that affect PICC occlusion rates in hospitalized patients.	Descriptive correlational study	**Maintenance:** -Flush the PICC lumens after each use and frequently if intravenous fluids are administered continuously.
Gupta et al. [36], 2021 Tunneled and routine peripherally inserted central catheters placement in adult and pediatric population: review, technical feasibility, and troubleshooting. USA	To discuss indications, contraindications, procedural techniques, imaging, routine care, and tunneled PICCs.	Literature review	**Pre-procedure:** -Perform a detailed review of the patient’s medical and surgical history. -Observe imaging exams before PICC placement. -Optimize the patient’s coagulation profile when a history of coagulopathy or anticoagulant/antiplatelet therapy is present. -Choose a smaller gauge needle to reduce the risk of bleeding. -Select alternative extremities in people who need repeated PICC placement. -Complete a preliminary assessment of the vasculature using US imaging. -Select the vein to be punctured: The basilic vein is always preferable for the initial access attempt; The brachial vein should be the second choice; The cephalic vein is the least indicated and is associated with a higher incidence of thrombosis. -Avoid placing the access in the antecubital fossa. -Select the smallest gauge PICC (catheter size should be less than 45% of the vein diameter). -Use antimicrobial PICC to reduce the risk of infection. -Use a peri-procedural checklist during PICC insertion. **During the procedure:** -Administer sedative therapy to adults with significant anxiety or cognitive impairment. -Perform the PICC insertion procedure under US or fluoroscopic guidance. -Adapt the length of the PICC to the patient and document the measurement used. -Fix the PICC with an adhesive stabilization device or by sutures. **Post-procedure:** -Confirm the positioning of the PICC tip at the cavo-atrial junction by fluoroscopy, intracavitary EKG, or chest X-ray. **Maintenance:** -Use heparin as a PICC-blocking solution. **Team management:** -Train the nursing team on how to handle and manage the PICC.
Krein et al. [14], 2019 Use of designated nurse PICC teams and CLABSI prevention practices among US hospitals: A survey-based study. USA	To identify the prevalence of designated nurse PICC teams and the factors associated with their existence among USA acute care hospitals.	Quantitative survey with a random sample	**Team management:** -Implement designated nurse PICC teams.
Broadhurst et al. [10], 2019 CVAA Occlusion Management Guideline for Central Venous Access Devices—2nd Edition. Canada	To review the published literature and develop a guideline for the management of CVAD occlusions.	Literature review	**Maintenance:** -Perform a chest X-ray to confirm the correct positioning of the PICC. -Strategies to prevent occlusion of the CVAD should be carried out routinely. -Flush the lumens using the pulsatile or “push-pause” technique with an appropriate amount of flushing solution. -Minimize the number of times the CVAD is accessed. -Flush with 0.9% sodium chloride between administering incompatible medicines and solutions due to precipitation or residues forming. -For solutions that are not compatible with 0.9% sodium chloride, use a compatible solution (e.g., dextrose in water) before and after administering the solution and medication, followed by flushing with 0.9% sodium chloride to clear the solution from the CVAD. -To maintain patency in specific clinical situations, consider using alternative solutions to block the lumens after flushing with 0.9% sodium chloride. **Team management:** -Ensure ongoing training and validation of the skills of health professionals responsible for the care and management of CVAD in the following areas: Principles of device permeabilization; Assessment, prevention, and management of catheter occlusion; Type of CVAD and additional characteristics; Documentation and continuous monitoring. -Measuring results and including information on the type of catheter, patient information, treatment details, and occlusion management methods.
Registered Nurses Association of Ontario [4], 2021 Vascular Access Best Practice Guideline—2nd Edition Canada	Provide evidence-based recommendations and resources for inserting, assessing, and maintaining CVADs.	Systematic Review	**Pre-procedure:** -Perform a systematic assessment of the patient before inserting a VAD. -Provide health education to patient and their families/caregivers about their CVAD. **During the procedure:** -Provide non-pharmacological and pharmacological pain management strategies during the insertion of a VAD. **Team management:** -Implement practical education sessions on the insertion and management of VADs. -Implement vascular access specialists or teams to support the insertion and management of VADs.

## Data Availability

The data presented in this study are available on request from the first author.
